# FurA-Dependent Microcystin Synthesis under Copper Stress in *Microcystis aeruginosa*

**DOI:** 10.3390/microorganisms8060832

**Published:** 2020-06-01

**Authors:** Yuanyuan Chen, Jiaojiao Yin, Jin Wei, Xuezhen Zhang

**Affiliations:** 1 Engineering Research Center of Green Development for Conventional Aquatic Biological Industry in the Yangtze River Economic Belt, Ministry of Education, College of Fisheries, Huazhong Agricultural University, Shizishan street 1, Wuhan 430070, China; yychen@jhun.edu.cn (Y.C.); JjYin123@163.com (J.Y.); weijin1027@hotmail.com (J.W.); 2 College of Life Science, Jianghan University, Wuhan 430070, China

**Keywords:** *Microcystis aeruginosa*, microcystins, copper, Fe–S cluster, *mcy*

## Abstract

Massive blooms of cyanobacteria frequently occur with microcystin (MC) production. Cyanobacteria are exposed to copper stresses such as copper algaecides which are often used to remove cyanobacterial blooms. However, copper increased the MC production of cyanobacteria, and the underlying mechanism remains unclear. The present study investigated the relationship between copper exposure (0.5 and 3 µM) and MC synthesis in *Microcystis aeruginosa* PCC 7806. The study concluded that the content of intracellular MCs increased by nearly two times both in 0.5 and 3 µM copper. High-throughput RNA sequencing (RNA-seq) provided evidence that copper mainly attacked Fe–S clusters, with evidence of changes in iron, sulfur, iron uptake regulators (fur), glutaredoxins and dehydratase genes. The transcription of numbers of genes implicated in iron uptake, MC synthesis and *furA* was also evaluated with quantitative real-time PCR (qRT-PCR). In these three Cu treatment groups, the amount of MCs increased as copper elevated. As the expression of *mcyD* gene was directly regulated by FurA and copper ions affected the expression of the FurA-related genes, we believed that MC synthesis genes were controlled by copper. This study has made a further understanding of the mechanism of the increase in MC synthesis of *M. aeruginosa* PCC 7806 treated with copper-based algaecides. We aimed to understand the mechanism of copper ion influencing the synthesis of MCs.

## 1. Introduction

Colony formation of *Microcystis*, a bloom-forming cyanobacteria, is closely associated with algal blooms in eutrophic freshwater systems worldwide. *Microcystis* blooms have been more and more common in a variety of water bodies and become a worldwide problem. Many intracellular metabolites, such as algae organic matters (AOMs) and microcystins (MCs), can be produced by some species of *Microcystis* in blooms. Intracellular metabolites are released during the growth and lysis of *Microcystis* cells. It has been proved that AOMs can be used as precursors to various carcinogenic disinfection by-products (DBP) [1,2]. MCs are highly toxic to the liver and cause severe liver disease [3]. Certain concentrations of MCs can induce tumorigenesis [4] and are associated with neurological diseases [5]. Therefore, various operative methods were used to prevent or reduce these water quality problems derived from these cyanobacteria (e.g., CuSO_4_, H_2_O_2_, potassium permanganate, chlorine dioxide).

Copper-based algaecides were the most famous algaecides because of their highly toxic properties to algae and their low price [6,7]. Copper metal ions have been demonstrated to significantly affect the production of MCs [8,9,10]. However, little is known about the mechanism of how copper ions induce *Microcystis aeruginosa* (*M. aeruginosa*) cells to produce more MCs. Studies have shown that the release of MCs is mainly due to the loss of algal cell membrane integrity caused by copper exposure [8,10,11,12,13].

At present, the impact of copper on MCs mainly focuses on one aspect: the dynamics of intracellular and extracellular MCs when cyanobacteria cells are dosed with copper. Fan et al. studied the treatment after MC release from copper exposures [14,15]. The risks of soluble MCs in water compared to total MCs after treatment with copper were explored [16,17]. Finally, the majority of studies are concerned the relationship among the concentrations of MCs in water environment, cellular MC concentrations and copper ion concentrations. There were sigmoidal relationships among copper doses and algal responses such as cell density, photosynthesis and cell membrane integrity [18]. Tsai concluded that with the increase of copper sulfate exposure concentration, the extracellular MC concentration gradually increased [13]. Although previous studies provided some evidence of a relationship between copper concentration and MC release, there is basically no research on the mechanisms of increased MC synthesis following treatment with copper-based algaecides.

The management and restoration of water quality after harmful algal blooms is challenging. The use of copper-based algaecides continues to be one of the most common measures to eradicate cyanobacterial blooms [7]. Therefore, it is foreseeable that cyanobacteria and copper contamination often occurs together in freshwater systems. Furthermore, most studies have demonstrated that copper metal ions have a significant influence on the growth and MC production of cyanobacterial cells within a certain concentration range [8,9,10]. However, copper increases the MC production of cyanobacteria, and the underlying mechanism remains unclear. Our aim is to understand the mechanism of copper ion influencing the synthesis of MCs. To this end, we considered the relationship between the levels of MCs and copper concentration, and conducted transcriptase sequencing of *M. aeruginosa* PCC 7806 exposed to different concentrations of copper (0, 0.5 and 3 µM). The studies revealed that the content of intracellular MCs increased by nearly two times both in 0.5 and 3 µM copper. High-throughput RNA sequencing (RNA-seq) provided evidence that copper mainly attacks Fe–S clusters, with evidence of changes in iron, sulfur, iron uptake regulators (fur), glutaredoxins and dehydratase genes. In conclusion, *M. aeruginosa* produces more MCs in a copper-dependent manner, which seems to be regulated by FurA, as copper excess leads to imbalances of intracellular iron metabolism by disturbing the assembly of iron–sulfur cofactors. FurA is necessary to obtain more iron to participate in the formation of iron–sulfur clusters, and regulates the expression of *mcyD*.

## 2. Materials and Methods

### 2.1. Strain and Culture Conditions

The axenic strain *M. aeruginosa* PCC 7806 was provided by The Institute of Hydrobiology, Chinese Academy of Sciences (Wuhan, China) and grown in BG11 media. The *M. aeruginosa* PCC 7806 strain was grown at 25 °C under continuous illumination (20 µmol photons m^−2^s^−1^). *M. aeruginosa* cells were cultured in 200 mL modified BG11 media with different concentrations of copper in 250 mL conical flasks that were acid-washed prior to use to avoid copper contamination.

### 2.2. Copper Exposures

A copper salt (CuSO_4_) was selected for this study and purchased from Sigma-Aldrich (St. Louis, MO, USA). We exposed the *M. aeruginosa* with a series of concentrations of copper. Before the initiation of toxicity experiments, 1 mM Cu stock solution was prepared from CuSO_4_ using ultrapure water obtained from a MILLI-Q ultrapure water purification system (Millipore, Bedford, MA, USA). Then, 300 mL of *M. aeruginosa* was placed in 500 mL beakers for cultivation.

In order to study the relationship between copper ion concentrations and the concentration of the *M. aeruginosa* solution (represented by OD680), we incubated the *M. aeruginosa* with different concentrations of copper (0, 0.1, 0.2, 0.3, 0.5, 1, 2, 3, 4 and 5 µM). Control groups did not receive copper additions. The experiments were replicated in triplicates. After 4 days of treatment, we measured the response of *M. aeruginosa* to CuSO_4_. The EC_50_ (4 days) of copper on *M. aeruginosa* was calculated by GraphPad Prism 5 (GraphPad Software, San Diego, CA, USA).

### 2.3. RNA Extraction and Reverse Transcription

The total RNA was extracted from 50 mL of *M. aeruginosa* cultures during the exponential growth phase using a Trizol reagent (TaKaRa, Dalian, China) according to the manufacturer’s protocol. One microgram of RNA was transformed into cDNA using the PrimeScriptTM RT reagent Kit from Takara.

### 2.4. M. aeruginosa Transcriptome Data Analyses

To perform transcriptome experiments, the *M. aeruginosa* PCC 7806 strain was grown in BG11 under normal conditions and under copper-filled conditions (addition of 0.5 and 3 μM CuSO_4_). RNA extraction, data capture and the analyses were performed by Novogene Bioinformatics Technology Co., Ltd. (Beijing, China), and a detailed description of this method can be found in the Supporting Information (Materials and methods; Lin 2–41). Differentially expressed genes were selected based on fold change > 2 and *p* value < 0.05. The data for each set of samples were analyzed and each set contained 3 biological replicates. The array data dealt with in this study were deposited in NCBI’s Gene Expression Omnibus and are accessible through GEO Series accession number GSE108380 (Available online: https://www.ncbi.nlm.nih.gov/geo/query/acc.cgi?acc=GSE108380).

### 2.5. Quantitative Real-Time PCR

*M. aeruginosa* PCC 7806 were exposed to 0.5, 3 μM copper and then the cells were collected at 0, 12, 24, 48 and 72 h for quantitative real-time PCR (RT-qPCR). RT-qPCR was used to test for relative expression levels of three microcystin synthetase genes (mcy) considered representative indicators (*mcyA*, *mcyD*, *mcyH*) and four genes related to the Fe–S cluster (*furA*, *furB*, *furC*). The specific primers used to amplify the genes involved in toxin synthesis and iron stress, including the housekeeper gene RNA polymerase subunit C (rpoC1), are shown in Table S1. RT-qPCR was conducted using the SYBR^®^ Green Premix Ex TaqTM II kit (Takara, Dalian, China). RT-qPCR specifications were 95 °C for 2 min, 40 cycles of 95 °C for 15 s and 60 °C for 30 s [19]. We used the 2^−ΔΔCt^ method described by Pfaffl to calculate the relative gene expression of the treated group cells which was expressed as the ratio of control gene expression [20].

### 2.6. Intracellular Copper and Iron Concentrations of M. aeruginosa

We added an additional 0.5 or 3 μM copper to BG11 and examined the intracellular copper and iron concentrations after 24, 48, 72 and 96 h of exposure. The cells were centrifuged at 10,000 *g* for 10 min and washed three times with ice-cold iron and copper-free BG11. We digested the cells with 10% nitric acid and 95 °C water baths for 3 h, until the liquid was clear. Intracellular copper and iron contents were analyzed by an inductively coupled plasma optical emission spectrometer (OPTIMA 8000DV, PerkinElmer, Waltham, MA, USA).

### 2.7. Gel Mobility Shift Assays

#### 2.7.1. Extraction of DNA

The samples of *M. aeruginosa* were harvested immediately and chromosomal DNA was obtained by a modification of a technique [21]. In brief, a 30 mL aliquot of mid-exponential-phase culture was harvested by centrifugation. The medium was removed and we used 500 mL of 5 mM EDTA (pH 8.0)-50 mM Tris-HCl (pH 8.0)-50 mM NaCl to resuspended the pellet. A 2 mg/mL final concentration of Lysozyme was added to the solution and incubated at 65 °C for 30 min. The solution was incubated at 55 °C for 40 min with 20 mL of 10% sodium dodecyl sulfate and 10 mL of proteinase K (10 mg/mL). An equal volume of 4 M ammonium acetate was added to the supernatant followed by extraction with phenol–chloroform–isoamyl alcohol (25:24:1). Lastly, 2 volumes of isopropanol were used to precipitate DNA.

A forward primer 5′-GTCGATCGCCCATGGCTGCCTAC-3′ and the reverse primer 5′-CAGTTGGGAATTCCCGCTTAGATG-3′ were used for the PCR amplification of the Fur gene sequence [22]. Amplification was carried out in a reaction mixture (50 mL) and incubated at 95 °C for 5 min followed by 40 cycles of 95 °C for 30 s, 58 °C for 1 min and 72 °C for 1 min.

#### 2.7.2. Overexpression and Purification of Fur Protein

The *furA* gene sequence obtained by PCR was digested with the restriction enzymes NcoI and EcoRI (Takara, Tokyo, Japan). The band was cloned into the expression vector, using a T4 DNA ligase (Takara, Japan). Then, the vector was transformed to *Escherichia coli* JM 109 cells (Promega, Madison, WI, USA) with the ligation product.

*E. coli* BL21 cells (Promega, Madison, WI, USA) were used to overexpress the recombinant Fur protein. Colonies containing the pET-28a (+)/fur plasmid could grow in Luria–Bertani medium kanamycin. Then, 0.8 mM concentration of isopropyl β-d-thiogalactoside (IPTG) was used to induce the *E. coli* BL21 cells expression. Cells were grown at 37 °C for 8 h. The expression level was monitored by sodium dodecyl sulfate-polyacrylamide gel electrophoresis (SDS-PAGE) electrophoresis.

The purified Fur protein were obtained using HisPur™ Cobalt Resin (Thermo Fisher Pierce, Waltham, MA, USA), according to the manufacturer’s instructions.

#### 2.7.3. Detect Protein–DNA Interactions

According to the method described by Martin-Luna et al., we cloned and overexpressed the *fur* gene and purified the recombinant protein [22]. Electrophoretic mobility shift assay (EMSA) was carried out using a LightShift Chemiluminescent EMSA kit (Pierce, Waltham, MA, USA) according to the manufacturer’s instructions. The primers p*fur*C 5′-GAGCTTAGGATGCCACACCC-3′ and p*fur*N 5′-CATAGTGTTAGAATCGACTTGG-3′ [23] were used for amplifying a fragment of 331 bp located upstream of the *mcyD* gene. The binding reaction was carried out at room temperature for 20 min with 50 ng/μL poly(dI-dC), and 10 mM EDTA in 1 × binding buffer (LightShift™ chemiluminescent EMSA kit, Pierce, Waltham, MA, USA) using 0.3 μM of *fur* protein and 20 fmol of biotin-end-labeled target DNA. As ferrous readily oxidizes to Fe^3+^in air, 5mM Mn^2+^ was used instead of ferrous iron. 

### 2.8. Determination of MCs

After the addition of different concentrations of copper (0, 0.5 and 3 µM), the *M. aeruginosa* cells were cultured for 7 days. Copper treated and untreated (control) *M. aeruginosa* biomasses were separated and harvested by centrifugation at 8000× *g* for 10 min. The supernatant obtained by centrifugation was used as the sample of extracellular microcystin. The method described by Zhou was used to extract intracellular microcystins [8]. In brief, the *M. aeruginosa* cells sediment was washed three times and then re-suspended in ultrapure water (Milli-Q, Billerica, MA, USA). The cells were subjected to three freeze/thawing cycles and then sonicated baths for 5 min prior to centrifuge separation and filtration. When we calculated the amount of intracellular microcystins, we used biomass as a standard. Intracellular and extracellular MC concentrations were assessed, according to the manufacturer’s specifications (Beacon, Portland, ME, USA).

An ELISA assay microplate kit for microcystins contained a standard solution of 0, 0.1, 0.3, 0.8, 1.0 and 2.0 μg/L microcystins, with the wells immobilized with antibodies. In the MC water sample analysis, a stop solution was utilized to test the settings. The treatment of the sample and the standard solutions for color development prior to photometric detection was performed according to the manufacturer’s recommendations. Each well contained 200 μL of sample volume for microcystin determination.

### 2.9. Statistical Analyses

Statistical analyses of the data were carried out using the SPSS 19.0 (SPSS, Chicago, IL, USA) and GraphPad Prism 5 (GraphPad Software, San Diego, CA, USA). In order to determine significant differences between the control and copper exposure groups, ANOVA (one-way analysis of variance) was adopted using a Tukey HSD. All data were analyzed using statistically significant differences defined by *p* < 0.05 and indicated by * for significance. All data were analyzed using at least 3 independent samples and expressed as the mean ± standard deviation.

## 3. Results

### 3.1. Copper Toxicity

**Figure 1:**
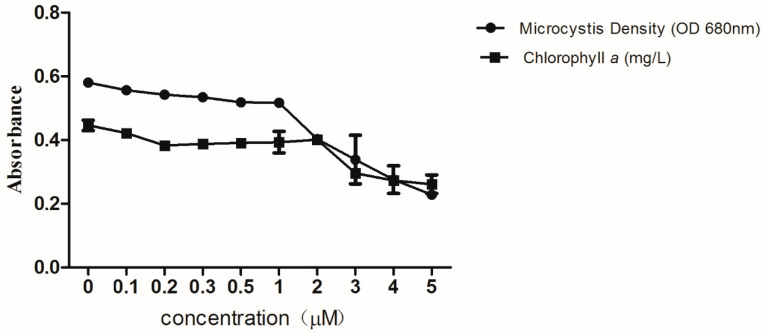
The algicidal ability of copper. Exponentially growing cells of *M. aeruginosa* PCC 7806 were diluted to OD 680 nm at 0.58 and cultured in a BG11-Cu medium supplemented with the copper concentration (0, 0.1, 0.2, 0.3, 1, 2, 3, 4 and 5 µM) for 4 days. The experiments were conducted in triplicates and the average with the standard deviation is plotted. * represents significance at *p* < 0.05 between the copper-treated groups and the control group.

We incubated *M. aeruginosa* cells with external copper (0, 0.1, 0.2, 0.3, 0.5, 1, 2, 3, 4 and 5 µM) for four days, to obtain the copper concentration required to inhibit the growth of *M. aeruginosa*. We observed a dose- and time-dependent inhibition of copper on the growth of *M. aeruginosa* from Optical Density (OD) 680 and chlorophyll *a* (Figure 1). EC_50_ represents the effective concentration at which half of the maximum response is observed. The EC_50_ 4 DAT for the decreases in OD 680 of *M. aeruginosa* was 2.99 µM, calculated using GraphPad Prism 5 (GraphPad Software, San Diego, CA, USA) (Table S2). According to this, we selected 0.5 µM (a weakly inhibitory concentration, Figure S1) and 3 µM copper for exposure to *M. aeruginosa* in our study.

### 3.2. Transcriptomic Responses to Low and High Copper 

To analyze the transcriptomic responses of *M. aeruginosa* following acute copper exposure, nine RNA libraries of RNA-Seq were constructed: 0 (grown in BG11 under normal conditions), 0.5 and 3 (with an addition of freshly prepared CuSO_4_) µM copper-treated groups, each treatment group with three replicates. The sequencing data is of good quality and does not require re-sequencing.

**Figure 2:**
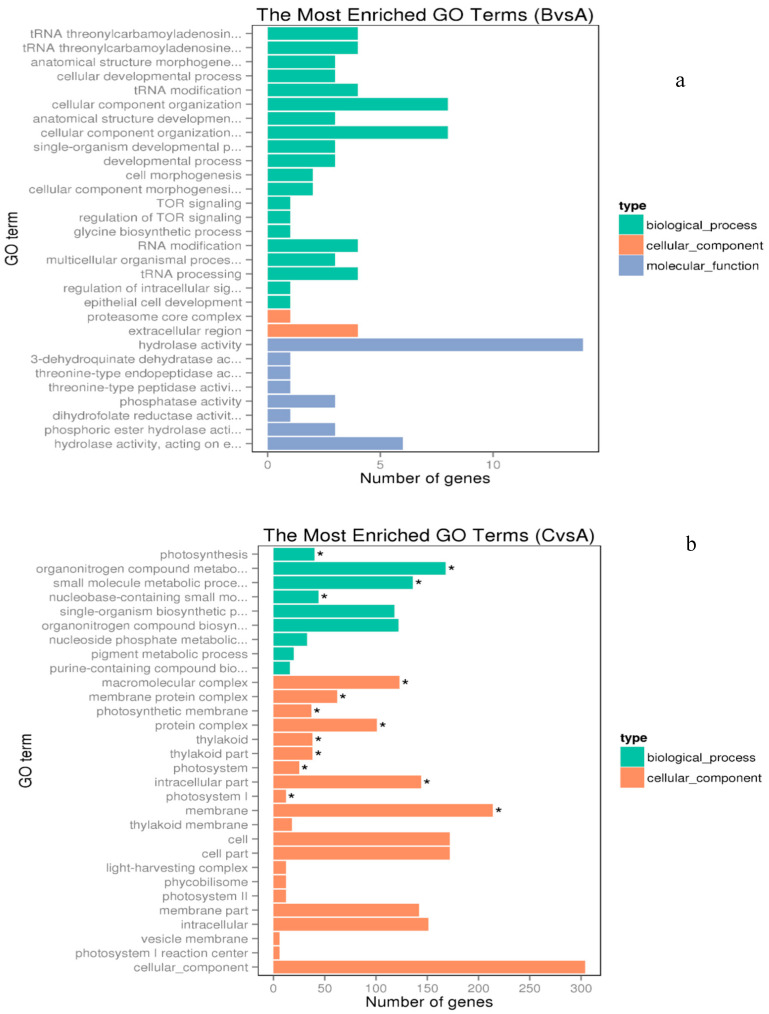
The significantly enriched Gene Ontology (GO) term of the 0.5 µM copper treatment group (**a**) and 3.0 µM copper treatment group (**b**). Green, orange and blue represent the enriched biological process, cellular component and molecular function. * represents significance at *p* < 0.05 between the copper-treated groups and the control group.

With fold change > 2 and *p* value < 0.05 criteria, in the 0.5 µM Cu treatment group, only 52 genes were differentially expressed. Among them, 32 genes were up-regulated and 20 genes were down-regulated (Figure S2). A total of 1306 differentially expressed genes (633 up-regulated and 673 down-regulated) were identified between the control and the 3 µM treated groups (fold change > 1.50, *p* < 0.05) (Figure S3). To understand the pathways and processes involved in the copper-mediated transcriptional responses of *M. aeruginosa*, the differentially expressed genes were further carried out by Gene Ontology (GO) and KEGG pathway analyses. In the 3 µM copper treatment group, the significantly enriched GO terms are shown in Figure 2b. However, there are no significant over-representations of GO terms or pathways (Figure 2a) in the 0.5 µM Cu treatment group, probably because there are few numbers of significantly differentially expressed genes.

### 3.3. Copper Stress Induces both Iron–Sulfur Cluster Biogenesis and Cluster Target Genes

From the GO term level, the data showed that the GO term involved in Fe–S cluster stabilization and biogenesis were significantly regulated (3 µM copper supplementation) (Table A1). From the gene level, the transcriptome studies conducted in the copper excess experimental group (3 µM copper supplementation) showed that the expression of iron, sulfur, glutaredoxin and dehydratase homeostasis genes of *M. aeruginosa* was significantly affected (Table A2). In addition, Fur-regulated genes which are commonly used to regulate high-affinity iron uptake, particularly MC synthetic, genes were generally regulated (Table A2). However, when a low copper stressed (0.5 µM) *M. aeruginosa* was compared with the control group, the genes that were significantly enriched in the 3 µM groups regulatory were not all found. Extensive effects on the expression of iron–sulfur cluster-associated pathways, such as cysteine biosynthesis, were shown in the copper stress (3 µM) transcriptome data (Table A2). Cysteine tRNA ligase that participates in cysteine metabolism were also significantly up-regulated. All of these data indicated that the biogenesis and/or repair of Fe/S clusters in *M. aeruginosa* were affected by copper.

### 3.4. Quantitative Real-Time PCR

#### 3.4.1. Iron Metabolism Genes under Copper Stress Conditions

In the experimental group of copper excess (0.5 and 3 µM copper supplementation), transcriptome studies of the *M. aeruginosa* PCC 7806 strain showed significant effects on MC synthesis and iron homeostasis gene expression. Fur-regulated genes for high-affinity iron uptake were generally regulated. To obtain accurate determinations, we used the *rpoC* gene as a housekeeper gene in all the RT-PCR analyses.

**Figure 3:**
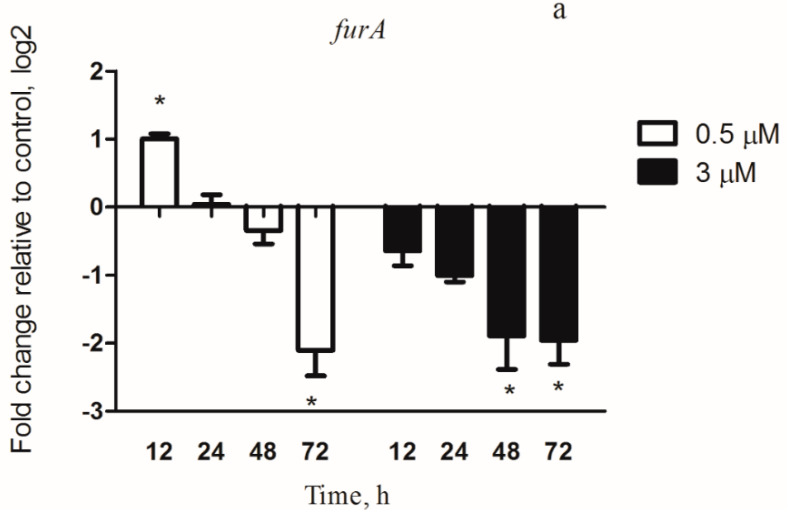

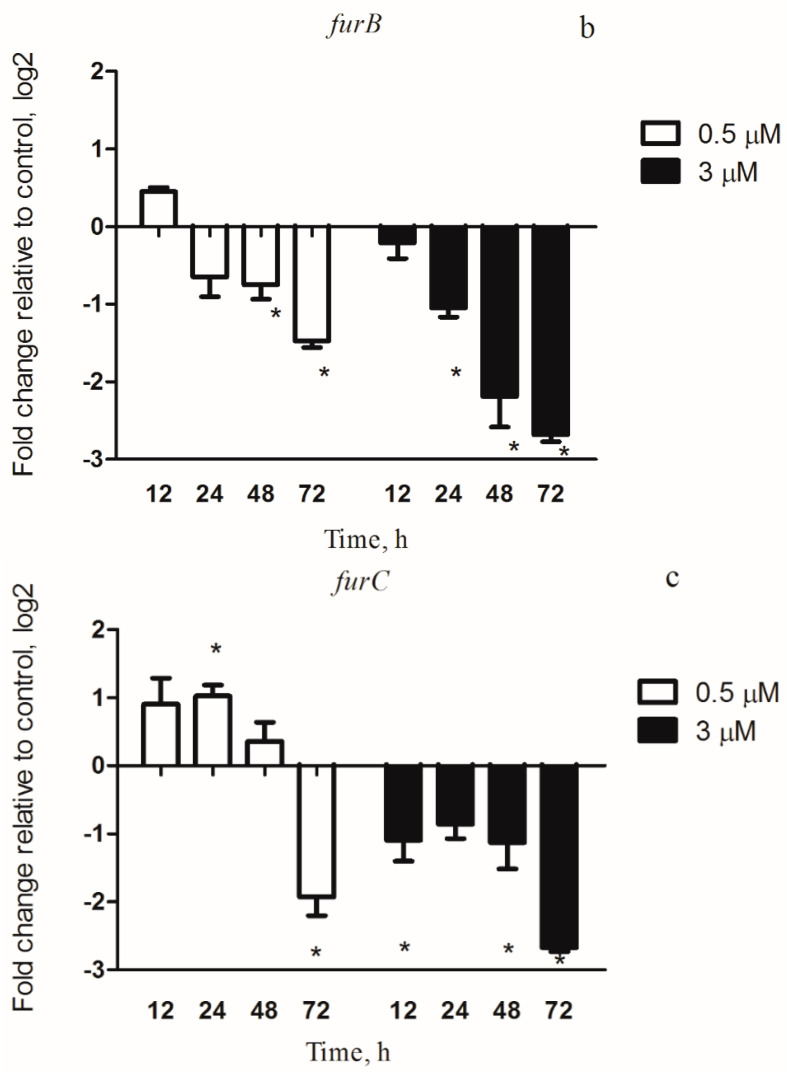
Relative expression levels of the iron metabolism genes (*furA*, *furB*, *furC*) in *M. aeruginosa* PCC 7806 under copper stress. (**a**) the *furA* gene; (**b**) the *furB* gene; and (**c**) the *furC* gene. The 0.5 µM: *M. aeruginosa* cells were incubated with 0.5 µM copper for 12, 24, 48, or 72 h. The 3 µM: *M. aeruginosa* cells were incubated with 3 µM copper for 12, 24, 48, or 72 h. Results are the mean ± SD from 3 separate determinations. * represents significance at *p* < 0.05 between the copper-treated groups and the control group.

The metalloregulator FurA in *M. aeruginosa* PCC 7806 can transcriptionally regulate the iron stress-inducible proteins [24], outer membrane transporter [25] and mcy cluster [23] expression. The *furA* transcription level was in a time-dependent manner (Figure 3a) in the 3 µM copper exposure experiment. A significant decrease in *furA* transcription level was observed after 3 µM copper exposure with increasing time. In the 0.5 µM copper treatment group, we observed a similar transcriptional trend of *furA* in PCC 7806 (Figure 3a). Notably, the transcription level of *furA* reached the highest value after 12 h exposure. At 0.5 and 3 µM copper, the transcription levels of *furA* were decreased with increasing time after 24 h exposure.

The transcription of *furB* and *furC*, the other two Fur homologs, was also analyzed under conditions of copper stress. *furB* was a putative DNA-protecting protein and a sensor of oxidative stress in *M. aeruginosa* PCC 7806. The transcription profile of *furB* was significantly decreased with increasing time in the 3 µM copper group relative to the control group. At 0.5 µM copper, the *furB* transcription was slightly increased at 12 h, but was obviously downregulated after 24 h exposure with increasing time (Figure 3b).

At 0.5 µM copper, the transcript level of *furC* was increased in correlation with the exposure time and for up at 24 h compared with the untreated sample (*p* < 0.05). However, at 72 h, in 0.5 µM copper-treated samples, the *furC* levels were dramatically decreased. At 3 µM copper, the transcript level of *furC* was significantly decreased after copper incubation (Figure 3c).

#### 3.4.2. Non-Ribosomal Peptide Synthesis Genes under Copper Stress Conditions

**Figure 4:**
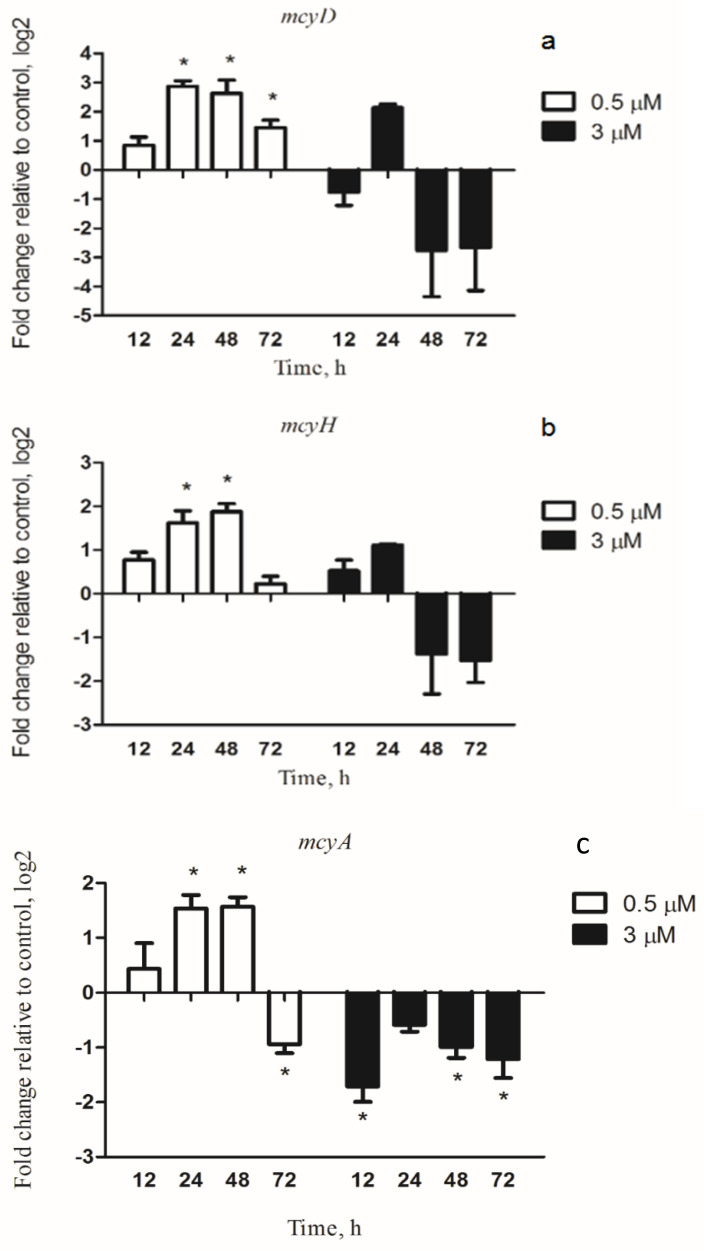
Relative expression levels of *mcyD*, *mcyH* and *mcyA* genes in *M. aeruginosa* PCC 7806 under copper stress. (**a**) the *mcyD* gene; (**b**) the *mcyH* gene. (**c**) the *mcyA* gene. The (0.5 µM) *M. aeruginosa* cells were incubated with 0.5 µM copper for 12, 24, 48, or 72 h. The (3 µM) *M. aeruginosa* cells were incubated with 3 µM copper for 12, 24, 48, or 72 h. Results are the mean ± SD from 3 separate determinations. * represents significance at *p* < 0.05 between copper-treated groups and the control group.

There are three genes, *mcyA*, *mcyD* and *mcyH*, in the *mcy* gene cluster. First, *mcyA* participates in the first step of MC synthesis. Furthermore, *mcyD* is involved in iron-responsive transcription [23,26]. Finally, studies have shown that *mcyH* is a gene that is critical for toxin production. The transcription of *mcyD* was significantly upregulated in *M. aeruginosa* incubated with 0.5 µM copper for up to 72 h and examined after 0, 12, 24, 48 and 72 h of exposure (Figure 4a). In contrast, the *M. aeruginosa* cells in 3 µM copper showed a less pronounced change in the *mcyD* transcript compared to the 0.5 µM copper-treated group. Although the transcription level of *mcyD* had slightly fluctuated in the 3 µM copper-treated group, no significant change occurred. The transcriptional change trend of *mcyD* in different copper concentration treatments of *M. aeruginosa* was related to the change of furA.

Similarly, the *mcyH* gene responded to the initial copper stress, and the transcription levels increased in a time-dependent manner at 0.5 µM copper compared with the untreated sample. However, at 3 µM copper, *M. aeruginosa* strain had slightly increased levels of *mcyH* transcript at 12 and 24 h. Then, there was a downward trend, but it did not change notably (Figure 4b). Expectedly, the transcription variation of *mcyA* was consistent with the trend of *mcyD* in both 0.5 and 3 µM copper, respectively (Figure 4c). A significant increase was observed, after 72 h exposure at 0.5 µM copper. At 3 µM copper, the transcription profile of *mcyA* was completely reversed compared with 0.5 µM copper (Figure 4c). Obviously, the *mcyA* levels had a downward trend after 3 µM copper incubation.

### 3.5. Intracellular Copper and Iron Content

**Figure 5:**
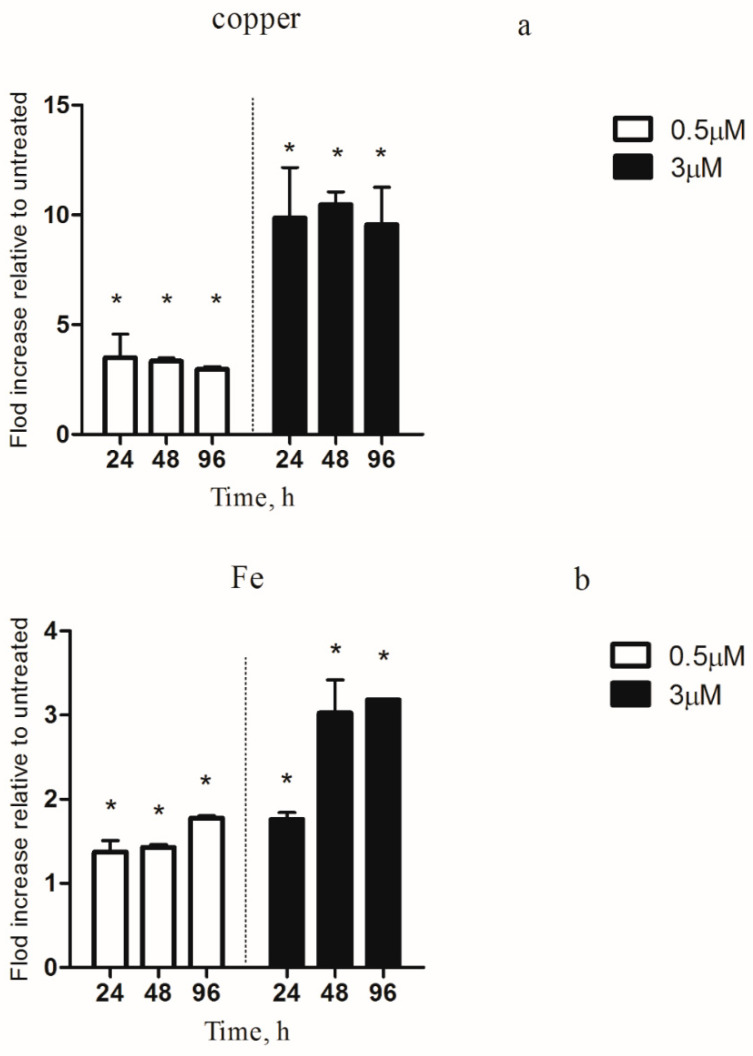
Copper and iron concentrations in *M. aeruginosa* PCC 7806. (**a**) The copper ion; (**b**) the iron ion. Intracellular copper iron ion concentrations were measured as described in Methods. Copper and iron concentrations are given relative to the untreated sample. The *M. aeruginosa* PCC 7806 was treated with copper (0.5 and 3 µM) for 24, 48, or 96 h. Y axis: The experiments were done in triplicates and the average with the standard deviation is plotted. * represents significance at *p* < 0.05 between copper-treated groups and the control group.

To test the overall content of intracellular copper, ICP measurements of the total cellular fraction were performed with cells grown under different copper concentrations. Similarities in intracellular copper levels were detected under copper excess (0.5 and 3 µM) and normal conditions (Figure 5a). In all of the copper overtreatment groups, the copper content in the *M. aeruginosa* cells increased significantly. At 0.5 µM copper, the total intracellular copper levels dramatically increased up to 3.5-fold compared to the untreated cultures and peaked at 24 h. The change of copper contents in 3 µM was similar to that of the 0.5 µM group. The copper content of the *M. aeruginosa* cells was increased by about 10-fold (Figure 5a).

The intracellular response of the iron concentrations to copper in *M. aeruginosa* was examined after 24, 48 and 96 h of exposure. The total intracellular iron content notably increased with increasing time (Figure 5b). The intracellular iron content was significantly increased by 1.4-fold in the 0.5 µM copper treated samples compared to the untreated cultures. In the 3 µM copper-treated samples, the intracellular iron concentrations were also significantly increased, and the same trend was observed with 0.5 µM copper (Figure 5b).

### 3.6. FurA Controls the Expression of McyD Gene

**Figure 6:**
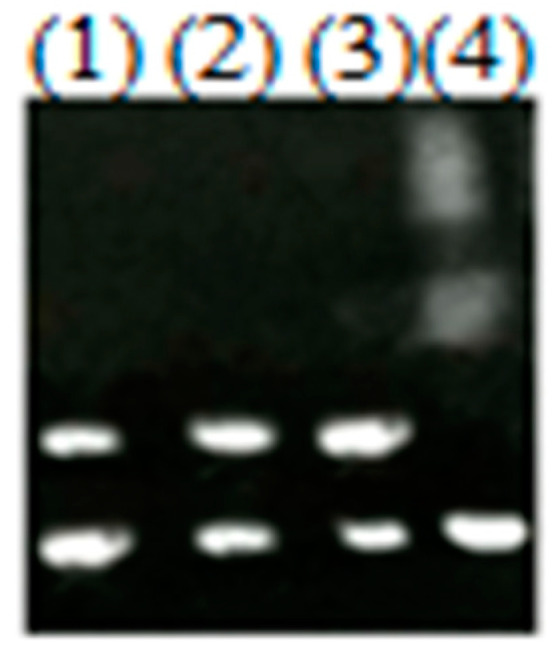
Electrophoretic mobility shift assays. Electrophoretic mobility shift assays showing the ability of FurA to bind in vitro to the promoter regions of *mcyD*. (1) The DNA fragments free. (2) The DNA fragments mixed with recombinant FurA protein at a concentration of 0.3 µM. (3) The DNA fragments mixed with Mn^2+^. (4) The DNA fragments and Mn^2+^ mixed with the recombinant FurA protein at a concentration of 0.3 µM.

Figure 6 shows the EMSA with a fragment using 0.3 µM Fur concentrations. Promoter fragments that specifically bind to the Fur are the upstream sequence of *mcyD*. Whereas the same concentrations of the FurA were unable to shift without Mn^2+^. As ferrous iron readily oxidizes to Fe^3+^ in the air, so Mn^2+^ was used instead of it.

### 3.7. MC Synthesis

**Figure 7:**
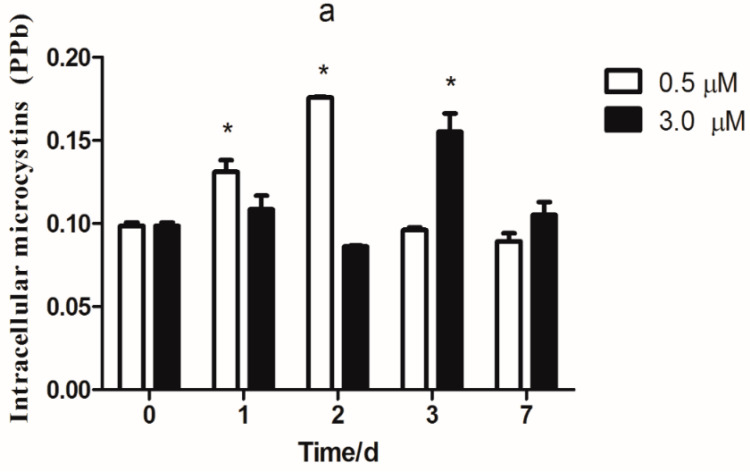

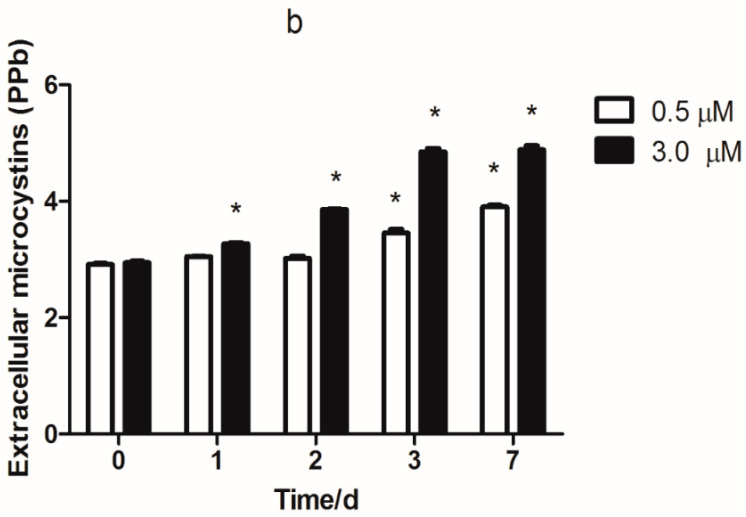
Intracellular and extracellular microcystins under copper stress (PPb). The levels of the intracellular (**a**) and extracellular (**b**) microcystin measurements in *M. aeruginosa* under conditions of copper excess (0.5 and 3 µM copper) for 1, 2, 3, or 7 days. The experiments were done in triplicate and the average with the standard deviation is plotted. * represents significance at *p* < 0.05 between copper-treated groups and the control group.

The intracellular and extracellular levels of MCs were measured under conditions of copper excess. We used biomass (Table S3, Chlorophyll *a*) as a standard to calculate the amount of intracellular MCs (total content of each MCs variant per unit of Chlorophyll *a*). In the 3 µM copper-treated samples, the total extracellular MC levels notably increased with increasing time (Figure 7b). The intracellular MC concentration was significantly increased after three days of exposure (Figure 7a). In the 0.5 µM copper-treated samples, the concentration of MCs notably increased as the exposure time increased from one to two days (Figure 7a). The results of extracellular MC concentrations are shown in Figure 7b. With the increase of exposure time, the concentration of MCs significantly increased in three and seven days.

## 4. Discussion

As few studies focus on the mechanisms of increased MC synthesis in *M. aeruginosa* PCC 7806 following treatment with copper-based algaecides, we aimed to understand the mechanism of copper ion influencing the synthesis of MCs. In this study, we analyzed the transcriptional changes of *M. aeruginosa* PCC 7806 strains during low- and high-copper concentration treatments. Both the minimal (0.5 µM) and the high (3 µM) copper treatments revealed the obvious induction of MC synthesis, mainly by regulating genes involved in energy metabolism and genes related to Fe–S clusters.

In our experiments, we found that one of the main targets of copper toxicity of *M. aeruginosa* PCC 7806 is the Fe–S cluster, the finding that has also been demonstrated in other microorganisms [27]. The homeostatic processes of iron and copper in living organisms are interdependent [27,28]. Transcription data on copper stress in the *M. aeruginosa* PCC 7806 strain showed how copper stress affects cell cytochemistry in its entirety, especially MC synthesis. Interestingly, we found that the pathways for obtaining iron and sulfur components in *M. aeruginosa* PCC 7806 were up-regulated, including the cysteine biosynthetic pathway and the MC synthesis pathway, all of which were dependent on Fur. Our results are consistent with previous studies on metal ion stress in *Bacillus subtilise* and *E. coli*. [28,29]. In addition, we found that some pathways that relied on the presence of iron–sulfur cofactors during copper stress in *M. aeruginosa* cells were up-regulated, such as those for cysteine biosynthesis (Table A2). On one hand, the copper stress affects the process of cluster assembly and transfer such as SufR, SufE, SufB (Table A2). However, on the other hand, copper also directly influences the stability of iron–sulfur clusters bound to the target proteins. In *Synechocystis* sp. PCC 6803, SufR (MAE_RS10070 in *M. aeruginosa* PCC 7806) is one of the transcriptional factors. Transcription factors that regulate gene expression response to the redox state of the bound Fe–S clusters serve as sensors for iron and cellular redox balance [30]. It can bind one [4Fe–4S] cluster per subunit and act as a repressor of the sufBCDS operon [31,32]. The operon, part of the SUF system, promotes the biosynthesis of Fe–S clusters in cyanobacteria and many non-photosynthetic bacteria [31,32,33,34]. Since the SUF system is an important system for the biogenesis of the iron–sulfur cluster, its apparent upregulation under copper stress also proved that the assembly of the initial process of the cluster was impaired.

The formation of iron–sulfur clusters is an important aspect of iron homeostasis. Fur mainly regulated the iron acquisition genes [24], and the regulation of Fur may affect other physiological effects associated with its metabolic function [30]. The Fur is one of the major regulators of iron levels in prokaryotic microorganisms [35]. Furthermore, it also affects genes involved in the energy metabolism, redox-stress resistance, DNA metabolism, TCA cycle, toxin production and other virulence factors [36,37,38]. It is therefore considered as a so-called master regulator. FurA regulates the transcription of many iron uptake genes and the mcy gene cluster promoter (*mcyD*) in *M. aeruginosa* PCC 7806 [23]. Hence, the link between iron stress caused by copper pressure and toxicity in *M. aeruginosa* can be explained by changes in the expression of *furA*. As expected, *furA* transcription decreased both at 0.5 and 3 µM copper (Figure 3a). We found that the copper toxicity increased with increasing exposure concentration (0.5 and 3 µM) (Figure 7a) and this trend was comparable to that of *furA*. However, *furA* transcription was reduced at 12 h. In addition to FurA, this may be mediated by other Fur homologs or transcriptional regulators such as NtcA.

Since FurA regulates the transcription of iron uptake proteins, we observed a significant increase in iron uptake in *M. aeruginosa* PCC 7806 (Figure 5b). Recent studies have found that iron stress affects the transcription of cyanobacteria *furB* [19]. In contrast, the transcription of *furB* was slightly affected under our experimental conditions. This may be due to the fact that the transcription of *furB* in *M. aeruginosa* cells during iron starvation is influenced by oxidative stress rather than iron stress [39]. The FurC protein contributes to the dimerization of Fur proteins. In our study, the transcription value of the *furC* gene was affected by the copper concentration. This may be because iron deficiency does not affect its transcription, but it may be involved in the oxidative stress protection of cells [39].

Despite that there have been numerous studies on the reaction of *furA* to iron deficiency [19,40]), knowledge on the regulating mechanisms of expression of the *mcyD* gene in cyanobacteria is limited. Fur directed the bidirectional *mcyDA* promoter, as illustrated in Figure 6. MCs might be a part of the set of responses to compete for copper stress. The presence of MCs may enhance the iron collection system’s response to iron deficiency, if it activates intracellular iron trappers, keeping Fur from DNA. The increase of MC synthesis in copper stress cells (Figure 7a) is partially related to the transcription of the toxin gene (*mcyD*, *mcyH*, *mcyA*) (Figure 4). In our experiment, a significant increase in intracellular MCs was observed under copper stress (0.5 µM), which is in agreement with the transcription of *mcyD* and *mcyH*. The relationship between *mcy* transcription and changes in MC content is consistent with previous studies by Sevilla [26] that toxin levels increase with increasing *mcyD* transcripts.

In Figure 7, the intracellular MCs increased significantly and then decreased in the low dose group, but in the high dose group, there was no significant change. The extracellular MCs significantly increased with elevated Cu doses and prolonged exposure time. These findings may be ascribed to two reasons. First, the addition of Cu led the living *M. aeruginosa* cells to produce more MCs and excrete intracellular MCs into water, thereby causing the increase of extracellular MCs in water and the increased or static state of intracellular MCs in *M. aeruginosa* cells. Second, the lysis and death of *M. aeruginosa* cells, due to the addition of excess Cu, led the intracellular MCs release into the water and thus increased the level of the extracellular MCs in the water. The observation agreed with the finding of Daly et al. and Zhou et al., who reported that the toxic *M. aeruginosa* kept their cyanotoxins within their cells, but dramatically released them into the surrounding water upon cell lysis [8,41]. What is different is that in our study, the intracellular MCs were constant or significantly increased. This is mainly due to the up-regulation of the *mcyD* expression. The *mcyD* encodes a polyketide synthase involved in microcystin synthesis in *M. aeruginosa* and is involved in iron-responsive transcription [26]. FurA regulates the transcription of the mcy gene cluster promoter (*mcyD*) in *M. aeruginosa* PCC 7806 [23].

In conclusion, the physiologically important pathways in *M. aeruginosa* PCC 7806 cells under the copper stress may be due to the changes of iron–sulfur cluster biogenesis. Furthermore, the downregulation of *furA* led to more MC production. *FurA* is necessary in order to obtain more iron to participate in the processes of iron–sulfur cluster destabilization and reconstitution. The production of MCs is controlled by copper, because the expression of *furA* is significantly affected by copper stress, and FurA regulates the expression of *mcyD*.

## Data Availability

The data used to support the findings of this study are included within the article.

## Supplementary Materials

The following are available online at https://www.mdpi.com/2076-2607/8/6/832/s1, Figure S1. The algicidal ability of copper (a) Growth curves and (b) the changes of Chlorophyll a content for M. aeruginosa cultured in BG11-Cu medium supplemented with the copper concentration (0, 0.5, and 3 µM) for 0, 12, 24, 48, 72, or 96 h. The experiments were done in triplicate and the average with standard deviation is plotted. Figure S2. The differentially expressed genes. control group: A; 0.5 µM: B. Figure S3. The differentially expressed genes. control group: A; 3.0 µM: C. Table S1. Sequences of the primers used in this study for *mcyD, mcyH, mcyA, furA, furB, furC, rpoC.* Table S2. *M. aeruginosa* responses to copper exposures: EC_50_, Slopes, and R^2^ values. EC_50_ values are presented as µM. R^2^ values represent goodness of fit of a sigmoidal. Table S3. *Microcystis* biomass under copper stress (mg/L).

## Appendix A

**Table A1:** Significantly GO term involved in Fe–S cluster stabilization and biogenesis under high-copper excess.

GO_accession	Description	Term_Type
GO:0051539	4 iron, 4 sulfur cluster binding	molecular_function
GO:0051536	iron–sulfur cluster binding	molecular_function
GO:0005381	iron ion transmembrane transporter activity	molecular_function
GO:0006826	iron ion transport	biological_process
GO:0044272	sulfur compound biosynthetic process	biological_process
GO:0015093	ferrous iron transmembrane transporter activity	molecular_function
GO:0015684	ferrous iron transport	biological_process
GO:0016730	oxidoreductase activity, acting on iron–sulfur proteins as donors	molecular_function
GO:0006790	sulfur compound metabolic process	biological_process
GO:1901681	sulfur compound binding	molecular_function
GO:0016667	oxidoreductase activity, acting on a sulfur group of donors	molecular_function
GO:0005506	iron ion binding	molecular_function
GO:0016226	iron–sulfur cluster assembly	biological_process
GO:0031163	metallo–sulfur cluster assembly	biological_process
GO:1901682	sulfur compound transmembrane transporter activity	molecular_function
GO:1901678	iron coordination entity transport	biological_process
GO:0016668	oxidoreductase activity, acting on a sulfur group of donors, NAD(P) as acceptor	molecular_function
GO:0016699	oxidoreductase activity, acting on hydrogen as donor, iron–sulfur protein as acceptor	molecular_function
GO:0006827	high-affinity iron ion transmembrane transport	biological_process
GO:0033573	high-affinity iron permease complex	cellular_component
GO:0034755	iron ion transmembrane transport	biological_process
GO:0006879	cellular iron ion homeostasis	biological_process
GO:0008199	ferric iron binding	molecular_function
GO:0055072	iron ion homeostasis	biological_process
GO:0008198	ferrous iron binding	molecular_function
GO:0072348	sulfur compound transport	biological_process
GO:0016671	oxidoreductase activity, acting on a sulfur group of donors, disulfide as acceptor	molecular_function
GO:0000097	sulfur amino acid biosynthetic process	biological_process
GO:0000096	sulfur amino acid metabolic process	biological_process
GO:0051537	2 iron, 2 sulfur cluster binding	molecular_function
GO:0010309	acireductone dioxygenase (iron(II)-requiring) activity	molecular_function
GO:0004089	carbonate dehydratase activity	molecular_function
GO:0008124	4-alpha-hydroxytetrahydrobiopterin dehydratase activity	molecular_function
GO:0003855	3-dehydroquinate dehydratase activity	molecular_function
GO:0004424	imidazoleglycerol-phosphate dehydratase activity	molecular_function
GO:0004664	prephenate dehydratase activity	molecular_function

**Table A2:** Selected genes induced in *M. aeruginosa* PCC 7806 after the high-copper treatment at 3 µM: the addition of freshly prepared 3 μM CuSO_4_.

	Locus Gene	Functional Description	3 µM
**Fe–S cluster response**	MAE_RS10070	transcriptional regulator sufR	7.06 × 10^−31^
	MAE_RS16045	cysteine desufuration protein SufE	0.00048425
	MAE_RS10065	Fe–S cluster assembly protein SufB	0.00015271
	MAE_RS00015	(2Fe–2S)-binding protein	0.00012458
	MAE_RS23065	(Fe–S)-binding protein	9.8206 × 10^−26^
	MAE_RS25860	photosystem I iron–sulfur center	0.001504
	MAE_RS06030	(2Fe–2S)-binding protein	0.0010832
	MAE_RS07140	ferredoxin:protochlorophyllide reductase (ATP-dependent) iron–sulfur ATP-binding protein	1.3806 × 10^−13^
	MAE_RS21970	4Fe-4S ferredoxin	1.6106 × 10^−8^
	MAE_RS24290	zinc/iron-chelating domain-containing protein	0.0035634
	MAE_RS10060	ABC transporter ATP-binding protein	0.0020487
	MAE_RS15100	glycine cleavage system protein T	0.0011042
**Iron**	MAE_RS00530	ferredoxin	0.0002315
	MAE_RS00890	ferredoxin	0.00014744
	MAE_RS06385	ferredoxin	1.1706 × 10^−8^
	MAE_RS04240	ferrochelatase	0.00043992
	MAE_RS05555	ferredoxin--NADP(+) reductase	6.7706 × 10^−10^
	MAE_RS10410	iron ABC transporter substrate-binding protein	7.2506 × 10^−5^
	MAE_RS13725	ferrous iron transporter B	3.7706 × 10^−34^
	MAE_RS13730	iron transporter FeoA	3.8906 × 10^−54^
	MAE_RS14865	ferrochelatase	9.9806 × 10^−7^
	MAE_RS18510	ferredoxin	0.0026747
	MAE_RS19620	iron ABC transporter substrate-binding protein	4.8806 × 10^−6^
	MAE_RS24680	Fe(3+) ABC transporter substrate-binding protein	1.1306 × 10^−9^
**Sulfur**	MAE_RS25765	sulfurtransferase	0.03116
	MAE_RS26150	sulfurtransferase DndC	5.5706 × 10^−5^
	MAE_RS22440	sulfurtransferase	0.0012032
	MAE_RS25470	IscS subfamily cysteine desulfurase	2.0906 × 10^−10^
	MAE_RS05935	cysteine desulfurase	0.019161
	MAE_RS06525	cysteine desulfurase	4.6006 × 10^−6^
	MAE_RS07485	molybdopterin synthase sulfur carrier subunit	1.6906 × 10^−6^
**Dehydratase**	MAE_RS07940	NAD-dependent dehydratase	0.0031625
	MAE_RS13050	GDP-mannose 4%2C6-dehydratase	9.9906 × 10^−6^
	MAE_RS13315	3-dehydroquinate dehydratase	3.0206 × 10^−8^
	MAE_RS17290	4a-hydroxytetrahydrobiopterin dehydratase	0.00011614
	MAE_RS20985	methylthioribulose 1-phosphate dehydratase	0.00015347
	MAE_RS21370	NAD-dependent dehydratase	7.8206 × 10^−6^
	MAE_RS25085	dTDP-glucose 4%2C6-dehydratase	0.0035724
	MAE_RS25915	imidazoleglycerol-phosphate dehydratase	2.6606 × 10^−7^
**Glutaredoxin 3**	MAE_RS01145	arsenate reductase%2C glutathione/glutaredoxin type	8.9406 × 10^−18^
	MAE_RS25430	glutaredoxin 3	0.0021661
**Microcystin**	MAE_RS16655	McyC protein	0.0000487
	MAE_RS16660	McyB protein	0.000000238
	MAE_RS16665	McyA protein	0.00011205
	MAE_RS16670	type I polyketide synthase(McyD)	5.5506 × 10^−21^
	MAE_RS16675	hybrid non-ribosomal peptide synthetase/type I polyketide synthase	3.0306 × 10^−13^
	MAE_RS16685	type I polyketide synthase	1.2406 × 10^−12^
	MAE_RS24610	non-ribosomal peptide synthetase	1.6306 × 10^−16^
	MAE_RS24620	non-ribosomal peptide synthetase	1.1206 × 10^−15^
	MAE_RS24650	non-ribosomal peptide synthetase	2.8506 × 10^−15^
	MAE_RS26180	non-ribosomal peptide synthetase	0.0000209
	MAE_RS26185	McnC protein	0.000000339
	MAE_RS26190	McnB protein	0.0000135
**Cysteine**	MAE_RS05935	cysteine desulfurase	0.019161
	MAE_RS06525	cysteine desulfurase	0.0000046
	MAE_RS12370	cysteine–tRNA ligase	2.1306 × 10^−8^
	MAE_RS16045	cysteine desufuration protein SufE	0.00048425
	MAE_RS18355	5-histidylcysteine sulfoxide synthase	0.000000907
	MAE_RS22460	cysteine synthase A	0.0038637
	MAE_RS25470	IscS subfamily cysteine desulfurase	2.0906 × 10^−10^
