# Supplementary material for: FurA-Dependent Microcystin Synthesis under Copper Stress in Microcystis aeruginosa
Source: Microorganisms. 2020 Jun 1;8(6):832. doi: 10.3390/microorganisms8060832 (PMC7356878; doi:10.3390/microorganisms8060832)
Supplement: Supplementary file 1 [file microorganisms-08-00832-s001.pdf]

Materials and methods

Microarray hybridization, bioinformatics and data analysis

RNA extraction and sequencing

To investigate the molecular mechanism, *M. aeruginosa* cells exposed to concentrations of 0.5 and 3 μM for 48 h were collected for transcriptomic analysis. For each concentration, there are three biological replicates. RNA extraction and sequencing was executed by Novogene Bioinformatics Technology Co., Ltd. (Beijing, China). Total RNA was extracted using the TRIzol reagent according to the manufacturer instructions and then genomic DNA was eliminated using DNase I (Invitrogen, New Jersey, NJ, USA). After the RNA samples detection, we used Ribo-zero kit to remove rRNA to enrich the mRNA. Then the mRNA was interrupted into short fragments in fragmentation buffer, and we use mRNA as template and hexahedron random primers to synthesize first strand of cDNA. Next, we synthesize the second-strand cDNA with dNTPs, DNA polymerase I and response buffer. We purify the double-stranded cDNA with AMPure XP beads. We used USER enzyme to degrade the second strand containing U cDNA. The purified double-stranded cDNA was subjected to terminal repair, followed by plus a tail and ligated sequencing linker, and then the AMPure XP beads were used for fragment size selection. At last, PCR amplification was performed and the PCR product was purified using AMPure XP beads to give the final library. After the library is constructed, Qubit2.0 is used to initialize the database. The library was diluted to 1 ng / μl, and then we use the Agilent 2100 to detect the insert size of the library. When the insert size is expected, the effective concentration

of the library was quantitatively determined by Q-PCR (effective concentration > 2 nM) to ensure the quality of the library. After the library was checked, the HiSeq / MiSeq sequencing was performed after pooling the different libraries according to the effective concentration and the target data.

Sequence tag preprocessing and mapping

We transfered the raw image data to the sequence data and saved it as FASTQ files. Performing quality control of alignment to determine if reordering was necessary. We clean the raw reads by clearing reads with the adapter, low sequence quality (> 30%), or a high percentage of unknown base (> 5%). Then we use SOA Paligner / SOAP2 software to map clean data to the annotated genome of the *M. aeruginosa* NIES-843 (https://www.ncbi.nlm.nih.gov/genome/?term=Microcystis+aeruginosa++NIES-843+) and allow up to 5 nucleotides to mismatch. Finally, the distribution of gene coverage is calculated using the alignment data. The gene expression levels were normalized as RPKM (reads per kilobase transcriptome per million mapped reads). In this study, differentially expressed genes were selected based on fold change > 2 (statistical power > 0.8) and P value < 0.05.

Gene Ontology and KEGG enrichment analysis

The software approach we used in the GO enrichment analysis was GOseq[1]. We usd KOBAS (2.0) to KEGG Pathway enrichment analysis.

Results

Fig. S1. The algicidal ability of copper

(a) Growth curves and (b) the changes of Chlorophyll *a* content for *M. aeruginosa* cultured in BG11-Cu medium supplemented with the copper concentration (0, 0.5, and 3 μM) for 0, 12, 24, 48, 72, or 96 h. The experiments were done in triplicate and the average with standard deviation is plotted.

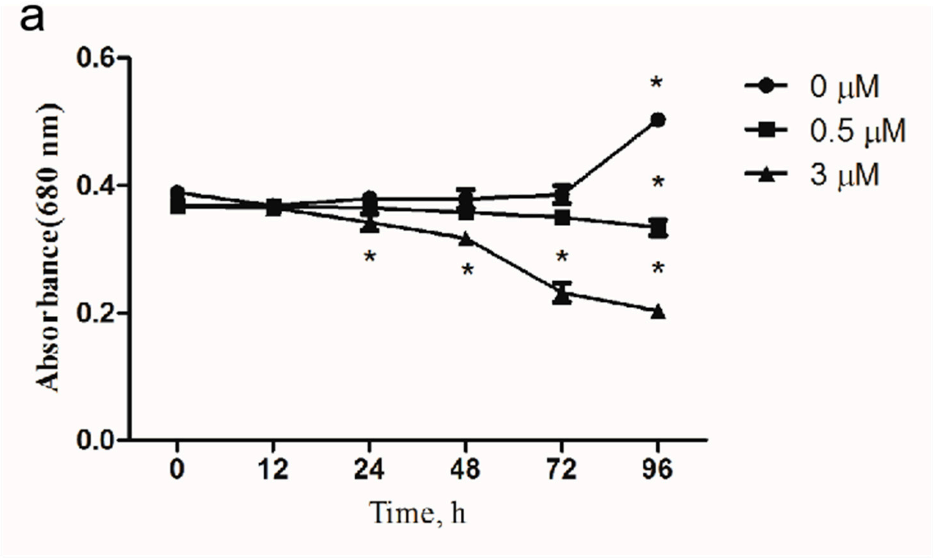

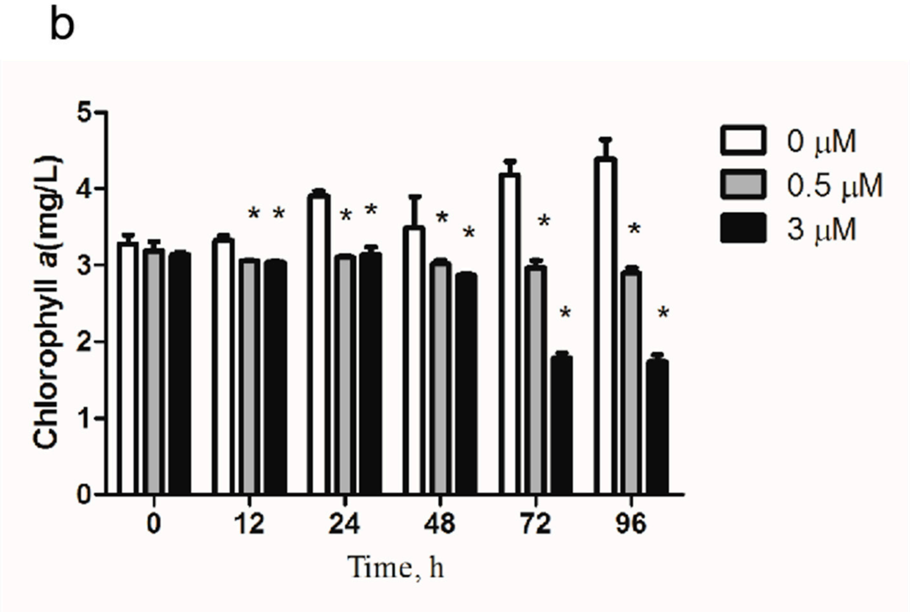

Fig. S2 The differentially expressed genes. control group: A; 0.5 μM: B.

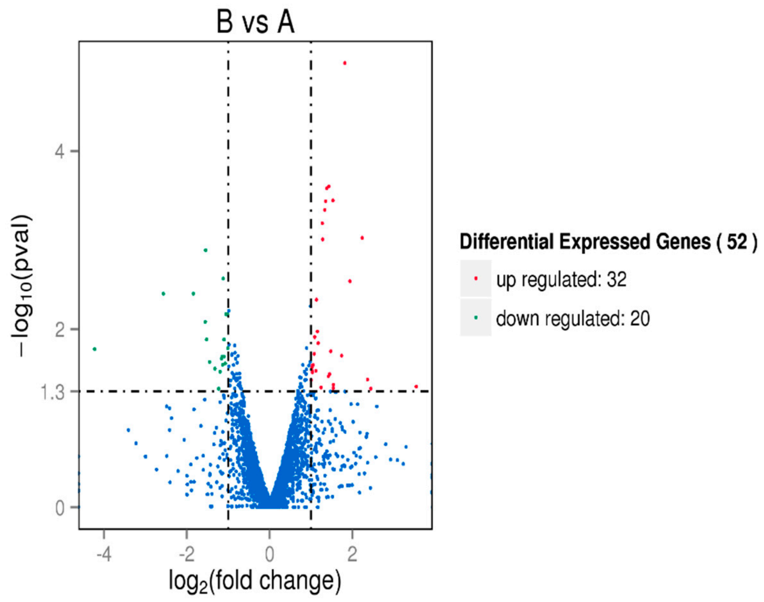

Fig. S3The differentially expressed genes. control group: A; 3.0 μM: C.

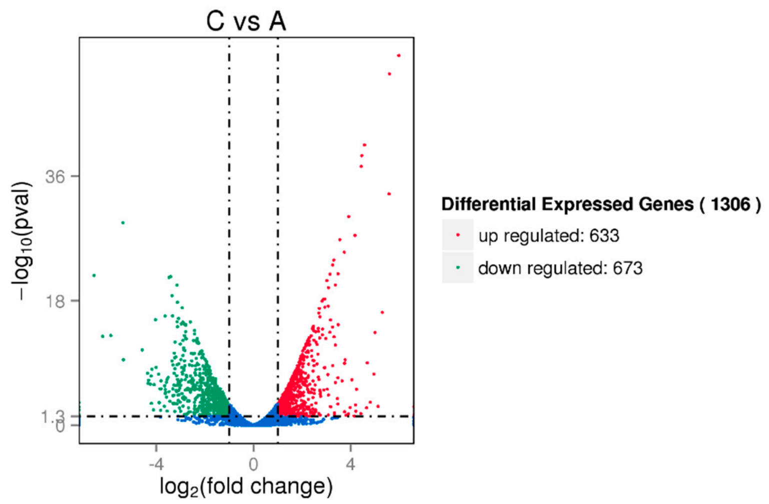

Table S1. Sequences of the primers used in this study for *mcyD, mcyH, mcyA, furA,*

furB, furC, rpoC

Primer	Sequence (5′–3′)	Target	Reference
mcyDF	gcatcttctaaagaaaagactcc	the first step of microcystin synthesis	This study
mcyDR	tattccccaagattgccataattt		
mcyHF	ttgtcttcgctccagcctat	Putative ABC transporter	Alexova et al. (2011)
mcyHR	ggccgacgaaaattcagata		
mcyAF	ttattccaagttgctcccca	Microcystin synthesis methyltransferase	Saker et al. (2005)
mcyAR	ggaaatactgcacaaccgag		
furAF	caatcatctcagtgccgaaga	Ferric uptake regulator	Alexova et al. (2011)
furAR	accctcggccaattccaact		
furBF	accttaaatcgggcagtatcc	Ferric uptake regulator	Alexova et al. (2011)
furBR	ctgcactgcaccctgtaattt		
furCF	gtgactgtgggaatcgctga	Ferric uptake regulator	Alexova et al. (2011)
furCR	aacacctatcagcgcgagaaa		
rpoCF	cctcagcgaagatcaatggt	RNA polymerase gamma subunit	Ginn and Neilan (2010)
rpoCR	ccgtttttgccccttacttt		

Given the source of each pair of primers. The functional class of each gene is also indicated.

Table. S2. *M. aeruginosa* responses to copper exposures: EC_{50}, Slopes, and R^2 values.

EC_{50} values are presented as μM. R^2 values represent goodness of fit of a sigmoidal.

		$CuSO_4$ 4 DAT
OD 680 nm	EC50 (95% CI)	2.99 (1.65, 5.42)
	Slope (95% CI)	1.99 (0.78, 3.21)
	R^2	0.99

Table S3. *Microcystis* biomass under copper stress (mg/L)

Exposure time (day)	0.5 μM	3.0 μM
0	0.9009±0.0003	0.98±0.0002
1	0.9008±0.0008	0.8206±0.0009
2	0.8442±0.0007	0.6988±0.0001
3	0.7956±0.0011	0.4103±0.0006
7	0.3023±0.0001	0.1288±0.0005

The levels of biomass measurements in *M. aeruginosa* under conditions of copper excess (0.5 and 3 μM copper) for 0, 1, 2, 3 or 7 days. The experiments were done in triplicate and the average with standard deviation is plotted.

Reference

1. Young, M. D., Wakefield, M. J., Smyth, G. K. & Oshlack, A. Gene ontology analysis for RNA-seq: accounting for selection bias. *Genome boil.* **11**, R14 (2010).
